# Breast Cancer Education and Empowerment in Ethiopia: Evaluating Community-Based Cancer Prevention Efforts Using the RE-AIM Framework

**DOI:** 10.1007/s13187-024-02453-6

**Published:** 2024-05-28

**Authors:** Breanne E. Lott, Sarah Yeo, Etsegenet Bekele, Firaol Birhanu, Rehima Hussein, Seada Muktar, Tsion Mengiste, Emebet Asfaw, Purnima Madhivanan, Biniyam Tefera Deressa

**Affiliations:** 1 Department of Epidemiology and Population Health, Albert Einstein College of Medicine, Bronx, New York, NY, USA; 2 Cancer Center, University of Arizona, Tucson, AZ, USA; 3 Tikur Anbessa Specialized Hospital, Addis Ababa University, Addis Ababa, Ethiopia; 4 Bridge The Gap Ethiopia, Adama, Ethiopia; 5 Mel and Enid Zuckerman College of Public Health, University of Arizona, Tucson, AZ, USA; 6 Adama Hospital Medical College, Adama, Ethiopia

**Keywords:** RE-AIM, Evaluation, Breast cancer, Awareness campaign, Screening, Ethiopia, Culture, Survivors, Knowledge

## Abstract

Cancer prevention challenges in Ethiopia include limited community awareness and low uptake of screening, which are in part driven by a lack of culturally and linguistically relevant cancer education appropriate for the diverse indigenous communities of this never-colonized nation. In 2022, a comprehensive multi-media breast cancer (BC) awareness campaign was implemented, featuring local cancer experts and survivors, with community-based screening events in the towns of Adama and Mojo. The RE-AIM framework was used to evaluate and describe its reach, effectiveness, adoption, implementation, and maintenance. Educational pamphlets, videos, social media posts, and interviews were distributed in person and through local and national media networks, reaching tens of millions of people and resulting in 525 individuals screened, with one diagnosis of early-stage cancer. During the free screening events, an interview-administered survey of BC knowledge, attitudes, and screening practices was conducted to inform future cancer education for this population. Among 287 survey respondents, about half correctly identified swelling (46%) or changing nipples/discharge (48.4%) as signs of BC. Maintenance challenges include the lack of a national screening program. Educational resources and a mobile app, translated into the local language, encourage continued patient empowerment to perform breast self-exams. In the absence of established BC prevention programs, “pop-up” mobile screening events can be effective for mobilizing communities to get screened. The paper highlights challenges and lessons gleaned from this community-based BC awareness campaign and screening event to inform future cancer education initiatives in Ethiopia and similar resource-limited settings.

## Introduction

Cancer incidence and mortality are growing rapidly, including for the hundreds of millions of indigenous peoples around the world, with cancer disparities driven by inequity in cancer prevention, screening, and treatment [1]. One review found that indigenous people tended to have less cancer knowledge and a higher propensity to refuse screening than non-indigenous people, highlighting the importance of tailored cancer education for improved cancer prevention in these populations [2].

The ethnic Oromo people are the largest indigenous population of Ethiopia, among approximately 80 socially, culturally, politically, and linguistically distinct ethnic groups [3, 4]. In this diverse setting, Ethiopia published its first National Cancer Control Plan in 2015—strategy one for promoting public awareness of cancer prevention called for the development of culturally acceptable messages, to be channeled through information networks such as health extension workers and the Health Development Army (volunteers that serve as intermediaries between the community and health system) [5]. Previous research has described cultural beliefs about cancer, such as it being a “curse from God/Allah” or caused by “*mich”* (ethnomedical cause of illness from a clash of cold and hot air) [6], yet health resources are not widely available to address these beliefs or deliver cancer information in local languages. Low community awareness persists as a major barrier to cancer prevention. Consequently, only 1.4% of women aged 40 and older have undergone a breast screening examination despite breast cancer (BC) being the most diagnosed malignancy [7]. In 2022, we conducted a multi-media BC awareness campaign in two languages, Afan Oromo (regional language) and Amharic (national language), featuring stories and lived experiences of Ethiopian cancer survivors. Skills were taught to encourage women to perform breast self-exams, and in two communities of the Oromia region, “pop-up” screening events provided free clinical breast exams. Here, we utilize the RE-AIM public health evaluation framework to describe these activities and lessons learned and to make recommendations for future cancer education in Ethiopia.

## Methods

### Setting and Intervention

In October 2022, a BC mass media awareness campaign was conducted with multiple dissemination strategies and outlets including social media, radio stations, and television platforms. Educational pamphlets, videos, and a digital application empowered community members to recognize the signs and symptoms of BC and to perform breast self-examinations. Media interviews with cancer survivors and experts addressed common myths and misperceptions, humanized BC through storytelling, and promoted screening. Additionally, cancer education and screening events were organized in the towns of Adama and Mojo, approximately 80 and 90 km southeast of the capital city, Addis Ababa. Free screenings, using clinical breast exams, were provided to eligible women aged 18 and above, regardless of screening history. All participants with palpable breast masses were followed up with ultrasound and additional diagnostic testing as appropriate at the local health facility. In the event of confirmed breast cancer, referral pathways existed to access surgery or chemotherapy treatment locally at Adama Hospital Medical College and radiotherapy at Tikur Anbessa Specialized Hospital in Addis Ababa. The program, consisting of the mass media campaign and two pop-up community-based screening events, was evaluated utilizing the RE-AIM framework [7]. Project monitoring records were reviewed to describe challenges and opportunities for cancer education and prevention. Ethical approval was obtained from the Adama Hospital Medical College Institutional Review Board.

### Framework and Measures

The RE-AIM framework was developed to evaluate community-based public health interventions and has been applied to various health promotion and disease management interventions by systematically examining five dimensions to determine public health impact: reach, effectiveness, adoption, implementation, and maintenance [7, 8].

### Reach

The project disseminated information about BC to the general Ethiopian community, including members of all genders and ages, while the screening events were targeted at adult women (≥ 18 years old) living in Adama and Mojo. Due to the nature of the intervention, accurately determining the specific number of individuals reached through the public outreach campaign proved challenging; estimates rely upon the best available information.

### Effectiveness

Effectiveness was assessed by documenting the number of individuals who participated in community-based screening events. Additionally, interview-administered surveys measured levels of knowledge, awareness, attitudes, and screening behaviors. The survey was developed by local investigators and delivered orally in Amharic and Afan Oromo, prior to in-person education and screening. Data were electronically captured through Google Forms on tablets at the screening events by trained data collectors. Descriptive statistics were analyzed using SPSS Version 29.

### Adoption, Implementation, and Maintenance

A narrative description of implementing partners, activities and resources in place to ensure the quality of implementation, and sustainability issues, such as training of health workers, provides additional context for the program and outcomes observed.

## Results

### Reach

Community-based education utilized social media, local and regional radio, national television (TV), pamphlets, and banners. The reach was substantial. Paid Facebook ads generated more than 16,000 engagements. A repost by the Hakim Facebook page, with more than 200,000 followers, amplified the impact. Radio broadcasts aired four times daily for a month and reached an estimated 30 million listeners. Four national TV interviews had a reach estimated to be on the magnitude of tens of millions. In Adama City, 30 strategically placed banners and educational videos displayed on digital billboards located in high-traffic areas and in banks engaged an estimated 100,000 viewers daily for 1 month. Approximately 3000 pamphlets were distributed in person by cancer survivors and health workers.

### Effectiveness

Outreach efforts in Adama and Mojo included a call-to-action to attend centrally located pop-up screening tents and resulted in a clinical screening of 525 individuals over the course of two Saturdays. Among those screened, 33 suspicious nodules were detected, prompting ultrasound screening. Upon referral, fine needle aspiration cytology (FNAC) was performed on 13 people, confirming one case of invasive cancer (Supplementary Fig. 1).

A subset of screening participants (*n* = 287) responded to the knowledge, attitudes, and practice (KAP) survey. Respondents were young (72.8% aged 20–40) and had at least high school–level education (92.7%), as detailed in Table 1. KAP survey responses are presented in Table 2. About half of the respondents correctly identified two queried signs of BC: swelling (46%) and changing nipples or discharge (48.4%). Cancer awareness came from the lay community (38.7%); traditional media such as TV, radio, or newspapers (34.1%); health professionals (18.1%); and social media (9.1%). Many women reported at least some awareness of breast self-examination (59.2%), and while many women were able to correctly identify aspects of self-examination, there was also some uncertainty. For example, many of those “aware” were unsure whether the exam should be performed in front of a mirror with hands raised (29.4%) or while lying down (31.8%), at what age the self-examination should start (17.1%), or whether the examination should include a check for swelling in armpits (19.4%). Almost all respondents agreed or strongly agreed that screening for BC is important (99.3%), that every woman should examine herself (98.2%), and that women should discuss it (96.9%). Still, 37% of women thought that self-examination was scary.

### Adoption

The community-based screening and education initiative garnered extensive support and adoption from various sectors, including governmental entities, non-governmental organizations (NGOs), and BC survivor groups. Essential contributions came from governmental bodies, such as the East Shoa Health Bureau, Adama Health Bureau, and Adama Hospital Medical College, which provided essential documents, facilitated connections, and contributed healthcare professionals and equipment. Both public and private healthcare institutions, including YOYA Hospital, the Ethiopian Blood Bank, and the Red Cross Society, actively participated by providing examination beds and furniture, as well as organizing blood donation efforts. Key NGOs, such as Bridge The Gap Ethiopia and the DEAR Foundation Switzerland, played a significant role in funding, creation and dissemination of educational materials, training of health providers, and event organization. Collaborative efforts were extended to partners such as the Ethiopian Society of Hematology and Oncology (ESHO) and Clinton Health Access Initiatives. A notable and unique aspect was the involvement of BC survivors as educators in the program. Additionally, 150 health professionals, including doctors, nurses, and health extension workers, received training on BC and were equipped to refer for or perform clinical breast exams; 50 professionals staffed the screening event, and an additional 100 were equipped to return to their own workplaces with new knowledge and skills.

### Implementation

The initiative was implemented by a structured team, with each member assigned specific roles. Key roles included logistics, training coordination, digital resource creation, and media engagement. Several implementation decisions were made to adapt to the cultural and health system context of Ethiopia. For example, the educational campaign invited interaction with the community. During the radio interview, a live question-and-answer segment featured open phone lines so that listeners could call in to ask questions to the oncologist (BTD) on air. In the absence of a national BC screening program, we designed the pop-up screening events as an implementation strategy for mobile delivery of screening (evidence-based practice). This community-based strategy was piloted as a way to overcome the implementation barrier that asymptomatic individuals who “feel well” are unlikely to seek preventive healthcare services, especially if care-seeking requires going to a hospital, a place “meant for the sick.” Large, white tents were rented and constructed in community centers, such as the stadium, to maximize visibility and accessibility. These white tents are frequently used in Ethiopia for weddings, funerals, and other community events, making them culturally acceptable places to gather. In addition, the tents could be configured however needed, with separate areas for registration, survey administration, group education, and private screening. The versatility of the physical space made mass screening feasible in a way that would not have been possible within the existing infrastructure of the local hospital.

### Maintenance

**Fig. 1:**
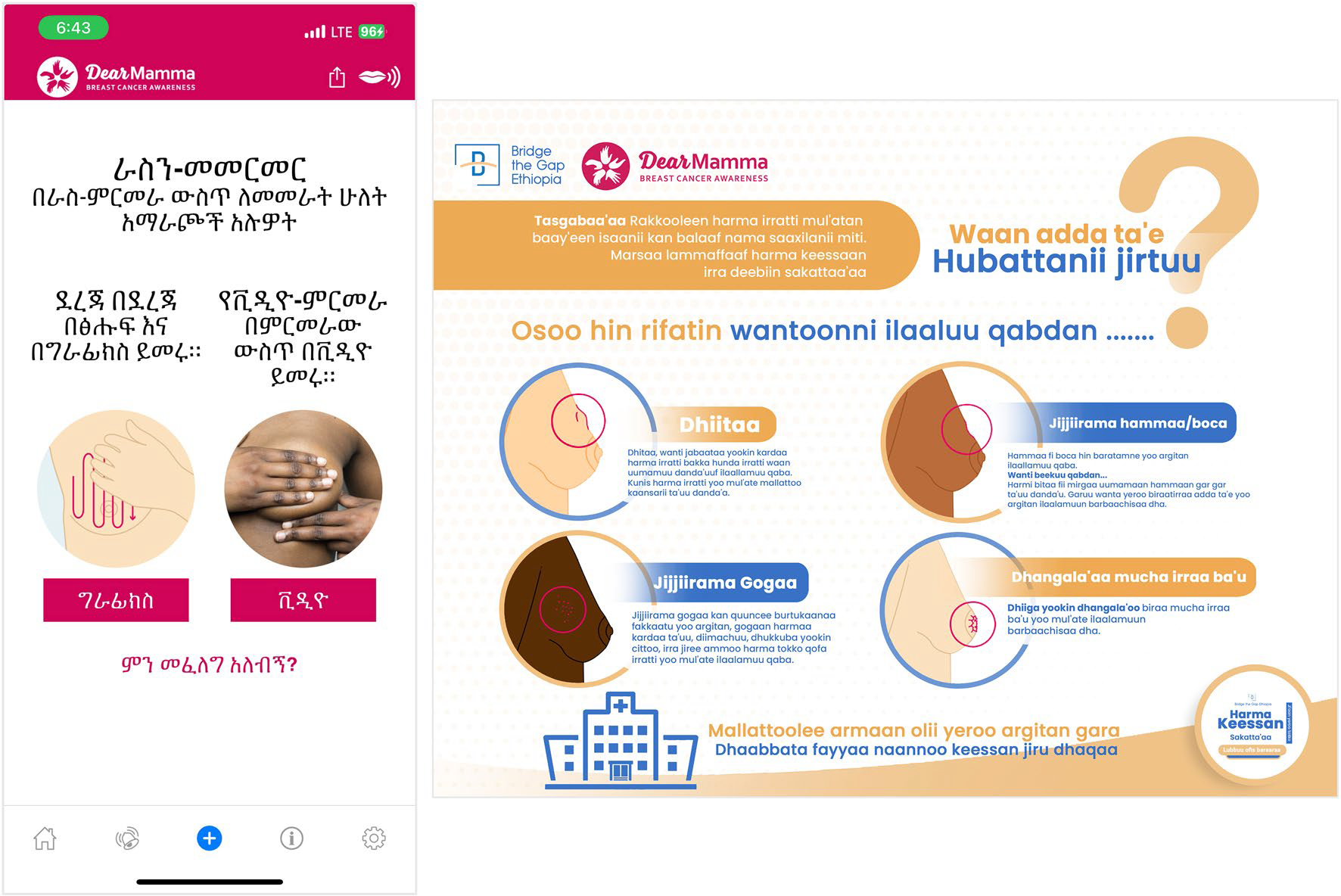
Educational materials and resources prepared in the local languages

While the awareness campaign and screening events were intended to be one-time activities, sustainability was considered through an emphasis on teaching the community to perform breast self-examinations. Women were empowered to continue monitoring their own breast health and to seek care if needed. One innovative component of the project was the translation of a mobile application (app) into Amharic and Afan Oromo. In collaboration with the DEAR Foundation Switzerland, the DearMamma Breast Cancer Awareness app was translated by health professionals and a demonstration of the app was included during education. Educational materials created (Fig. 1) and the training of health professionals, across cadres and health institutions, enhanced the knowledge and skills of the health workforce to continue practicing BC prevention and advocating for screening with their patients. Finally, the screening event presented an opportunity to explore the use of ultrasound technology, in combination with clinical breast exams, for routine screening in resource-limited settings where a mammogram is unavailable or inaccessible. Maintenance challenges include a lack of national training manuals or treatment guidelines, potentially impeding optimal care delivery or uniform implementation.

## Discussion

Community-based cancer education and screening initiatives have demonstrated effectiveness in raising cancer awareness and uptake of preventive behavior across settings in high- and low-income countries [9, 10]. In Ethiopia, such community-based programs may be especially important in connecting people with lifesaving cancer prevention services, especially in the absence of a national BC screening program. By providing the education and the screening (and follow-up diagnostic services) together in Adama and Mojo during a very short, focused educational campaign, the patient burden of screening is reduced by making clinical services available in an easy-to-access local setting with minimal travel required or wait times to be screened. The high level of community engagement we experienced, with more than 500 women screened over just 2 days, speaks to the feasibility and acceptability of this approach. In Ethiopia, mass media education has played an important role in combatting other diseases and represents an opportunity to increase cancer screening rates, just as it has done in other global settings [11, 12]. Specifically, radio has the power to reach rural and marginalized communities which is critical in a country where 77% of people reside in rural areas [13, 14]. With the growing accessibility of the Internet and social media, it has an emerging role in promoting preventive healthcare, such as cancer screening, and addressing health misinformation especially among younger audiences [15, 16]. As demonstrated by our screening participants, who reported many different sources of cancer information, a multi-modal approach to education should be used to deliver health messaging to different sectors of society. Unfortunately, for those who may have been motivated to screen by the educational campaign but who lived in communities other than Adama or Mojo, they likely faced difficulty finding and getting to such services, as these services are not yet available in the majority of Ethiopian healthcare facilities.

With regards to the cancer knowledge and attitudes of the population we surveyed, we must acknowledge the non-representative nature of the sample and the inability to extrapolate findings to a larger Ethiopian population as our respondents were highly motivated (and highly educated) individuals who were actively seeking BC screening (self-selection bias). Still, the findings can inform future BC education, such as addressing the fear of self-examination, something reported by almost 40% of our respondents. While we had planned to collect pre- and post-education surveys to measure change in knowledge and attitudes, we were unable to. High demand for screening necessitated that the event staff should only conduct the surveys at one point in time, prior to the in-person education and screening. As a result, we were unable to evaluate the effectiveness of our education.

A lesson affirmed through this process is that utilizing survivor voices to create engaging content, dispel myths, and humanize cancer proved to be very useful. Survivor testimonials emerged as potent tools for conveying information and fostering engagement as “survivors’ voices are the loudest.” In Ethiopia, where cancer is a highly stigmatizing disease, using first-hand survivor narratives stood out as a noteworthy best practice, with community members and policymakers commenting on the weight of the messages when delivered by actual cancer survivors. Their lived experiences deeply resonated with a diverse audience. Notably, this approach not only encourages individuals to participate in cancer screening but also effectively challenges the prevailing notion of cancer as an inevitable death sentence in Ethiopia. Drawing from these experiences, we strongly recommend the training of survivors in accurate cancer information and enabling them to assume the role of educators within their communities. This strategic approach not only aligns with established global best practices but also harbors the potential to yield substantial improvements in cancer outcomes in Ethiopia and other countries with low cancer awareness and high stigma [17, 18].

This program was implemented in collaboration with partners at different levels, indicating positive potential for adoption and maintenance. As a result of this program, culturally appropriate materials and mobile apps were developed and translated into the local language, providing ongoing utility, and healthcare professionals were trained and encouraged to advance BC prevention and detection. This type of community-based mass screening event may be an important adaptation of mobile screening that is appropriate for the context and resources available in Ethiopia. Future directions may include further investigation into pop-up cancer screening events, especially for rural communities where access to health facilities may be more limited and for indigenous populations who may be hesitant to screen in health facilities. One strength of this approach that couples community-wide education with a pop-up screening event is that it does not overburden the health system. Of the hundreds of women screened through this initiative, only 33 needed any follow-up diagnostic services (6.3%), and only one required treatment (0.2%). This ensures that medical resources are directed to those who need them while clearing individuals at low risk of BC. Another area for continued BC awareness may be engaging men. Population-based cancer data from Addis Ababa suggests that approximately 6% of BC cases occur in males, while in some Ethiopian health facilities, up to 18% of BC patients may be male [19, 20]. Aside from their personal risk for BC, the engagement of male partners and family members could play supportive roles for women seeking preventive cancer screening.

## Conclusion

A community-based BC awareness campaign and screening event, described using the RE-AIM framework, was successful in reaching a sizeable audience with culturally and linguistically appropriate cancer education and prevention services. The multi-modal education dissemination, using mass media, social media, and survivor voices, responded to the needs of a diverse population including rural indigenous Ethiopians. The uptake of screening at mobile mass screening events demonstrates the utility, feasibility, and acceptability of this implementation strategy. Ongoing adaptation and scaling of this service delivery approach may enhance cancer prevention in resource-constrained settings, with further research needed on changes to cancer awareness and screening practices over time.

## Supplementary Material

Supplementary file 1

## Figures and Tables

**Table 1:** Demographic characteristics of respondents to cancer awareness questionnaire

Characteristic	*N* (*n* = 287)	%

Age (years)		
20–30	126	43.9
31–40	83	28.9
41–50	54	18.8
51 +	24	8.4
Marital status		
Single	72	25.1
Married	186	64.8
Divorced	19	6.6
Widowed	10	3.5
Education		
Unable to read and write	14	4.9
Able to read and write (informally educated)	7	2.4
High School (grades 9–12)	128	44.6
Diploma and above	138	48.1
Occupation		
Housewife	98	34.1
Civil servant	90	31.4
Private	75	26.1
Student	24	8.4
Known family history of breast cancer		
Yes	22	7.7
No	265	92.3
If yes, who? (*n* = 22)		
Sister	4	18.2
Mother	4	18.2
Grandmother	2	9.1
Aunt	5	22.7
Other	6	27.3
Missing	1	4.5

**Table 2:** Breast cancer awareness, attitudes, and practices among survey respondents

Question	Response	*N*	%

Signs and symptoms of breast cancer			
Can swelling of the breast be a sign of cancer?	Yes	132	46.0
	No/I do not know	155	54.0
Can changing nipples or discharge be a sign of cancer?	Yes	139	48.4
	No/I do not know	143	49.8
	Missing	5	1.7
Screening awareness			
Do you know about breast cancer screening?	Yes	139	48.4
	No/I do not know	148	51.2
From which source have you heard about breast cancer screening?	Media (TV, radio, newspaper)	98	34.1
	Health professional	52	18.1
	In the community/ local people	111	38.7
	Social media	26	9.1
Do you know about breast self-examination?	Yes	170	59.2
	No/I do not know	117	40.8
If yes, should self-examination start at age 20? (*n* = 170)	Yes	133	78.2
	No	8	4.7
	I do not know	29	17.1
If yes, should the self-exam be performed standing in front of a mirror with hands raised?	Yes	118	69.4
	No	2	1.2
	I do not know	50	29.4
If yes, can the self-exam be performed lying down?	Yes	112	65.9
	No	4	2.4
	I do not know	54	31.8
If yes, can swelling or skin changes be seen by moving one’s fingers over the breast?	Yes	138	81.2
	No	7	4.1
	I do not know	25	14.7
If yes, can discharge be observed by touching the nipple?	Yes	141	82.9
	No	29	17.1
	I do not know	0	0
	Missing	1	0.6
If yes, should self-exam include a check for swelling in the armpit?	Yes	131	77.1
	No	7	4.1
	I do not know	33	19.4
Attitudes			
Do you think screening for breast cancer is important?	Strongly Agree	245	85.4
	Agree	40	13.9
	Neither agree nor disagree	2	0.7
	Disagree	0	0
	Strongly disagree	0	0
Should every woman self-examine her breast?	Strongly Agree	203	70.7
	Agree	79	27.5
	Neither agree nor disagree	2	0.7
	Disagree	3	1.0
	Strongly disagree	0	0
Should women discuss breast self-examination?	Strongly Agree	188	65.5
	Agree	90	31.4
	Neither agree nor disagree	5	1.7
	Disagree	4	1.4
	Strongly disagree	0	0
Do you think self-examination is embarrassing?	Strongly Agree	20	7.0
	Agree	8	2.8
	Neither agree nor disagree	5	1.7
	Disagree	192	66.9
	Strongly disagree	62	21.6
Is breast self-examination scary?	Strongly Agree	59	20.6
	Agree	47	16.4
	Neither agree nor disagree	129	45.0
	Disagree	50	17.4
	Strongly disagree	2	0.7
Practice			
Do you practice self-examination for breast changes?	Yes	218	76.0
	No	69	24.0

## Data Availability

The data that support the findings of this study are available from the corresponding author upon reasonable request.
