# Supplementary figures and images for: Breast Cancer Education and Empowerment in Ethiopia: Evaluating Community-Based Cancer Prevention Efforts Using the RE-AIM Framework
Source: J Cancer Educ. 2024 May 28;39(6):698–705. doi: 10.1007/s13187-024-02453-6 (PMC11568955; doi:10.1007/s13187-024-02453-6)

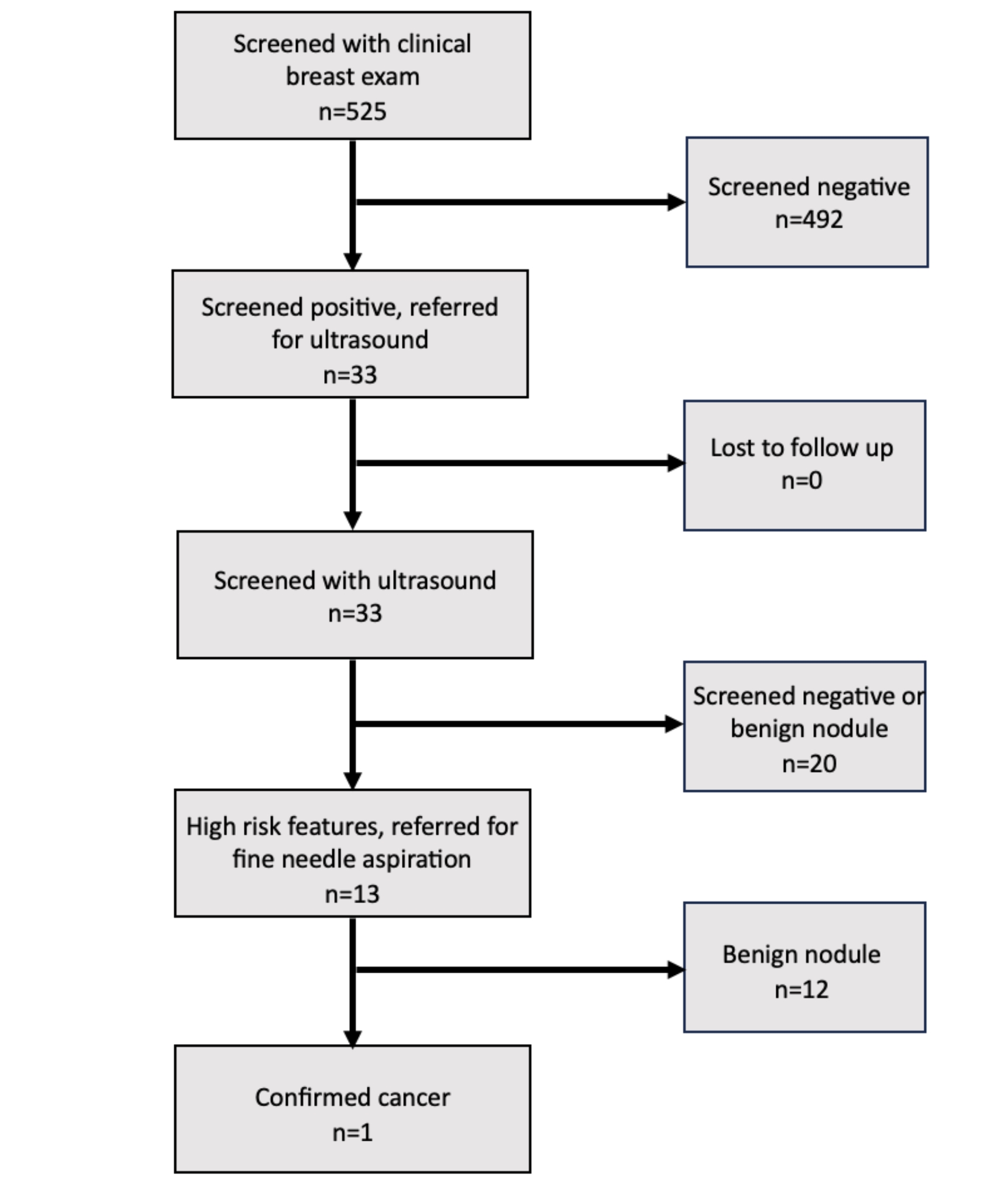

Supplement: Supplementary file 1 — Supplementary file1 (JPEG 294 KB) [file 13187_2024_2453_MOESM1_ESM.jpeg]
